# The effectiveness of an orthogeriatric service in Ain Shams University, Egypt: a quality improvement study

**DOI:** 10.1007/s11657-022-01144-3

**Published:** 2022-07-27

**Authors:** Heba G. Saber, Menna A. E. G. Aly

**Affiliations:** Geriatrics and Gerontology Department, Faculty of Medicine, Ain Shams University, Abbassia, Cairo, 11591 Egypt

**Keywords:** Orthogeriatric service, Length of stay, Osteoporotic fractures, Quality of life, Quality improvement

## Abstract

***Summary*:**

This quality improvement study assessed the effectiveness of an orthogeriatric service regarding fracture care and outcomes in terms of time to surgery, length of hospital stay, postoperative pain score improvement, depression and treatment decisions. The findings showed a significant reduction in time to surgery and mean length of stay following the implementation of orthogeriatric services (OGS).

**Introduction:**

Osteoporosis is a metabolic bone disease prevalent amongst the elderly, more commonly females, and puts them at increased risk of fragility fractures. OGS are recommended as a model of best practice for primary and secondary fracture care.

**Methods:**

This quality improvement study, conducted in our facility at Ain Shams University Hospital, Cairo, aimed to determine the effectiveness of an orthogeriatric service. We compared fracture care and outcomes before and after the implementation of OGS in terms of time to surgery, length of hospital stay, degree of postoperative pain score improvement, depression and treatment decisions. We included 128 patients aged 60 and above presenting to the emergency department with a fracture.

**Results:**

We found a significant reduction in the median time to surgery in the post-OGS group (*p* < 0.001) and a significant decrease in the mean length of stay in favour of the post-OGS group (*p* < 0.001). However, no significant difference was found between the two groups regarding the number of patients treated operatively, degree of postoperative pain improvement or susceptibility to depression.

**Conclusion:**

Since the orthogeriatric service began, preliminary data have been encouraging, with significant reductions in time to surgery and length of stay. This along with preoperative medical optimisation and collaborative discharge recommendations has improved overall patient outcomes even though more research is needed.

## Introduction

Specialised geriatric services are still a relatively new concept in the Middle East and North Africa. However, with the increasing percentage of the population ‘greying’, the importance of dedicated geriatric health services is being recognised. According to CAPMAS, 6.9% of the Egyptian population are over 60 and this is projected to rise to 10.9% in 2026 [1].

At the forefront of chronic health issues is osteoporosis. Osteoporosis is a metabolic bone disease prevalent amongst the elderly, more commonly females, and puts them at increased risk of fragility fractures [2]. Known fracture sites include the hip, wrist, ankle, shoulder and vertebrae, with hip fractures the most widely studied. This can be attributed to the significant impact on function and quality of life [3]. The ‘scorecard for osteoporosis in Europe’, or SCOPE, indicates that one-third of women and one-sixth of men will have an osteoporotic fracture during their lives [4]. Even though there is a lack of accurate data on the incidence of fragility fractures in the Middle East, it is understood to be worse than in other parts of the world, for example, Europe, where orthogeriatric services (OGS) are well established [5].

According to the British Geriatric Society’s Blue Book, optimum fracture care should include precipitous surgical admission with as little delay as possible, early postoperative mobilisation and multi-disciplinary rehabilitation. Secondary prevention should be initiated as well as fall risk assessment [6]. In fact, the Capture the Fracture® campaign by the International Osteoporosis Foundation (IOF) was developed to promote a certain standard of care for patients with low-impact fractures and lessen the occurrence of secondary fractures [7].

In accordance with the international movement towards co-management of older fracture patients, the first Egyptian OGS was started in Ain Shams University Hospital, Cairo. The Ain Shams OGS aims to provide pre- and postoperative services to fractured elderly and secondary prevention via a fracture liaison service with a dedicated outpatient clinic. Prior to this, there was no operationally structured OGS but rather a case-by-case approach (i.e. a surgeon would consult a geriatrician when a problem arose that could hinder surgery or when a postoperative complication occurred, most commonly postoperative delirium).

Using a quality improvement study design, the aim of this work was to determine the effectiveness of implementation of primary fracture care provided by the newly established orthogeriatric services in Ain Shams University Hospitals in terms of decision to undergo operative treatment, time to surgery, length of stay, degree of postoperative pain improvement and possibility of depression.

## Methods

### Study design

The study compared pre- and post-fracture care and outcomes before and after the implementation of the orthogeriatric services at Ain Shams University Hospital.

### Pre-implementation assessment (pre-OGS) stage 1

An assessment was done by the geriatricians in the OGS team as a part of a review of the actual situation at the facility in order to identify weaknesses that would need to be overcome when beginning to implement the OGS programme. This included 64 older adults (male and female, aged over 60) presenting to the emergency department (ED) with a fracture (low- or high-energy) and willing to participate in the study. The data collected included age, gender, pre- and postoperative pain scores by rating scale (0 = no pain and 10 = worst pain imaginable), length of stay, time to surgery, depression screening by the Patient Health Questionnaire (PHQ) [8], whether they underwent surgery or not and the type of fracture. Patients were enrolled between 1.1. and 31.5.2019 (5 months).

### Post-implementation assessment (post-OGS) stage 2

The comparison included a prospective study of 64 older patients presenting to the ED with fractures and willing to participate in the study. They were followed from admission until discharge with the newly founded OGS protocol. The same outcomes as above were collected but with the aid and intervention of the OGS team whenever appropriate. Patients were enrolled between 1.7. and 31.12.2019 (6 months). All participants were counselled on appropriate lifestyle behaviours to prevent secondary fractures as suggested by the National Osteoporosis Foundation (NOF) guidelines [9], assessed for recurrent fall risk and advised to follow-up with our clinic post-discharge.

### Ain Shams orthogeriatric service

The OGS protocol was set in January 2019 after a meeting with the heads of the Orthopaedic Surgery, Anaesthesiology and Geriatric Medicine departments. The geriatric team is responsible for the assessment of older patients in the ED along with the anaesthesiology and orthopaedic teams, and decisions for possible treatment options are made collaboratively.

The OGS is a mobile service provided on surgical wards; it is not a separate orthogeriatric ward. The service also includes an outpatient clinic dedicated to primary and secondary prevention of osteoporosis as well as fall risk assessment. All postoperative patients are advised to attend follow-up visits at the clinic. All recommendations provided by the OGS and the associated clinic follow the guidance of the NOF [9]. Screening for secondary causes of osteoporosis is performed as well as referral for bone mineral density measurement when indicated. Vitamin D and calcium intake are reviewed, and supplements, if needed, are recommended after assessment of serum levels. Fall risk assessment is also carried out and, accordingly, the patient is provided with counselling and lifestyle modification advice. The need for regular weight-bearing and muscle strengthening exercise is also addressed. Physiotherapy referral for those with mobility issues and that require supervised exercise is also done. Pharmacotherapy when needed for osteoporosis is provided as well as treatment follow-up.

After the initial ED assessment during the preoperative phase, daily ward rounds by consultant geriatricians and senior registrars are done for medical and surgical optimisation. A comprehensive assessment is performed that includes screening for common problems amongst elderly fracture patients like delirium, uncontrolled pain, depression, pressure ulcers and malnutrition. A medication review is also undertaken. Immediately after surgery, the geriatric team is responsible for daily follow-up of the patients, implementing mobilisation strategies, formulating the discharge plan and providing recommendations along with the Orthopaedic Surgery team.

The main target of the OGS team is to improve the health outcomes of older individuals presenting to the ED with fractures as measured by decreased time to surgery and length of stay, and screening and managing depression, delirium, malnutrition and risk of recurrent falls.

### Participants

The study sample comprised 128 men and women aged over 60 and presenting to the ED with fractures. Group A were the 64 patients before implementation of the OGS protocol and Group B comprised the 64 patients after implementing the OGS protocol.

Sample size was calculated using G*power programme, setting the type-1 error (*α*) at 0.05 and the power (1-*β*) at 0.9. Results from a previous study [10] showed that the mean inpatient hospital stay was 8.16 ± 3.75 days in the general medicine group compared to 6.75 ± 2.25 days in the co-management group. Calculation according to these values produced a sample size of 65 cases per group (130 total), taking into account a 10% drop-out rate.

### Outcomes

The primary outcomes included time to surgery, length of stay, degree of postoperative pain improvement, susceptibility to depression and treatment decision.

### Analysis

Analysis and interpretation of the present study obeyed the Standards for Quality Improvement Reporting Excellence (SQUIRE) guidelines [11].

We applied a pre–post design to evaluate the effectiveness of implementation of the OGS protocol in terms of treatment decisions, time to surgery, length of stay, pre- and postoperative pain scores and possibility of depression. We compared the same outcomes of interest in patients from the pre-OGS group to patients in the post-OGS group.

The collected data were revised, coded, tabulated and introduced to a PC using Statistical package for Social Science (SPSS 20). Data were presented and suitable analyses performed according to the type of data obtained for each parameter.

Descriptive statistics:

1. Mean, standard deviation (± SD) and range for parametric numerical data; median and interquartile range (IQR) for non-parametric numerical data.
2. Frequency and percentage of non-numerical data.

Analytical statistics:

1. Student’s *t*-test was used to assess the statistical significance of the difference between two study group means.
2. The Mann–Whitney test (*U*-test) was used to assess the statistical significance of the difference of a non-parametric variable between the two study groups.
3. The chi-square test was used to examine the relationship between two qualitative variables.
4. Fisher’s exact test was used to examine the relationship between two qualitative variables when the expected count is less than 5 in more than 20% of cells.

## Results

### Pre-OGS analysis (Group A)

Sixty-four patients over the age of 60 were included in the study. Males comprised 26.6% of the sample and females 73.4%. The mean age of the men (± SD) was 67 ± 8.12 years and of the women 68.63 ± 6.94. The mean preoperative numerical pain score (± SD) was 6.5 ± 2.3 and the mean postoperative numerical pain score (± SD) was 3.3 ± 2. The mean degree of postoperative pain improvement was 3.26 (degree of improvement was determined by the difference between pre- and postoperative pain scores). Fifty percent (50%) of patients were positive for depression when screened by PHQ2. The types of fractures included hip (50%), femur shaft (6.2%), distal femur (1.6%), proximal humerus (18.8%), ankle (12.5%) and distal radius (10.9%). In this group, 84.4% of the patients underwent surgery and 15.6% did not, either because they were deemed unfit for surgery or a conservative treatment option was considered more appropriate. The median time to surgery was 7 days (IQR 6–10) and the mean length of stay (± SD) was 13.8 ± 5 (Tables 1 and 2). Table 3 reports the choice of treatment for each fracture type.

**Table 1:** Comparison between Groups A and B regarding the studied variables

	Group A Pre-OGS		Group B Post-OGS		*t-*test
	Mean	SD	Mean	SD	*p* value
Age (years)					
Male	67	8.12	66.90	5.83	0.94
Female	68.63	6.94	70.26	7.88	0.22
Preoperative numerical pain score	6.5	2.3	7.4	1.6	0.012
Postoperative numerical pain score	3.3	2.0	4.2	2.0	0.012
Length of stay	13.8	5.0	7.0	3.9	< 0.001
	Median	IQR	Median	IQR	Mann–Whitney
Time to surgery	7	6–10	1	0–4	< 0.001
Improvement of postoperative pain scores	3	0–6	3	0–7	0.32

*OGS*, orthogeriatric service; *IQR*, interquartile range

**Table 2:** Comparison between Groups A and B regarding gender, depression, surgical decision and fracture site

		Group A		Group B		Chi-square test
		*N*	%	*N*	%	*p* value
Sex	Male	17	26.6	30	46.9	0.018
	Female	47	73.4	34	53.1	
PHQ	No	32	50	40	62.5	0.179
	Yes	32	50	23	35.9	
Surgery	No	12	18.2	10	15.6	0.812
	Yes	54	81.8	54	84.4	
Hip fracture	No	32	50	13	20.3	0.001
	Yes	32	50	51	79.7	
Femur shaft	No	60	93.8	60	93.8	1
	Yes	4	6.2	4	6.2	
Distal femur	No	63	98.4	63	98.4	1
	Yes	1	1.6	1	1.6	
Proximal humerus	No	52	81.2	62	96.9	0.005
	Yes	12	18.8	2	3.1	
Ankle fracture	No	56	87.5	59	92.2	0.380
	Yes	8	12.5	5	7.8	
Distal radius	No	57	89.1	61	95.3	0.188
	Yes	7	10.9	3	4.7	

*PHQ*, Patient Health Questionnaire

**Table 3:** Treatment choice for each fracture type in Groups A and B

	Group A				Group B			
	Conservative		Operative		Conservative		Operative	
	*N*	%	*N*	%	*N*	%	*N*	%
Ankle fracture	1	12.5	7	87.5	1	20.0	4	80.0
Hip fracture	7	21.9	25	78.1	8	15.7	43	84.3
Femur shaft	1	25.0	3	75.0	2	50.0	2	50.0
Distal femur	0	0.0	1	100.0	0	0.0	1	100.0
Distal radius	0	0.0	7	100.0	0	0.0	3	100.0
Proximal humerus	1	8.3	11	91.7	1	8.3	11	91.7

### Post-OGS analysis (Group B)

Sixty-four patients over the age of 60 were included in the study. Males comprised 46.9% of the sample and females 53.1%. The mean age of the men (± SD) was 66.9 ± 5.83 years and of the women 70.26 ± 7.88. The mean preoperative numerical pain score (± SD) was 7.4 ± 1.6 and the mean postoperative numerical pain score (± SD) was 4.2 ± 2. The mean degree of postoperative pain improvement was 3.26. When screened for depression using PHQ2, 35.9% of patients were positive. The types of fractures included hip (79.7%), femur shaft (6.2%), distal femur (1.6%), proximal humerus (3.1%), ankle (7.8%) and distal radius (4.7%). In this group, 81.8% of the patients underwent surgery and 18.2% did not, either because they were deemed unfit for surgery or a conservative treatment option was considered more appropriate. The median time to surgery was 1 day (IQR 0–4) and the mean length of stay (± SD) was 7 ± 3.9 (Tables 1 and 2). Table 3 reports the choice of treatment for each fracture type.

### Effectiveness of implementing the OGS protocol: pre–post OGS comparison

Regarding patients’ characteristics, no significant difference was found between the two groups regarding age (*p* = 0.688), distal radial fracture (*p* = 0.188) and ankle fracture (*p* 0.38). Significantly more patients in pre-OGS Group A presented with fractures of the proximal humerus (*p* = 0.005) as opposed to hip fractures, which were more common in post-OGS Group B (*p* = 0.001) (Table 2).

The two groups were compared regarding the chosen parameters and outcomes, which revealed the following.

The prevalence of depression as determined by screening with PHQ2 (*p* = 0.179) did not significantly change and neither did the degree of improvement of postoperative pain (*p* = 0.32). There was also no significant difference between the two groups in the number of patients that underwent operative treatment (*p* = 0.812) (Tables 1 and 2).

**Fig. 1:**
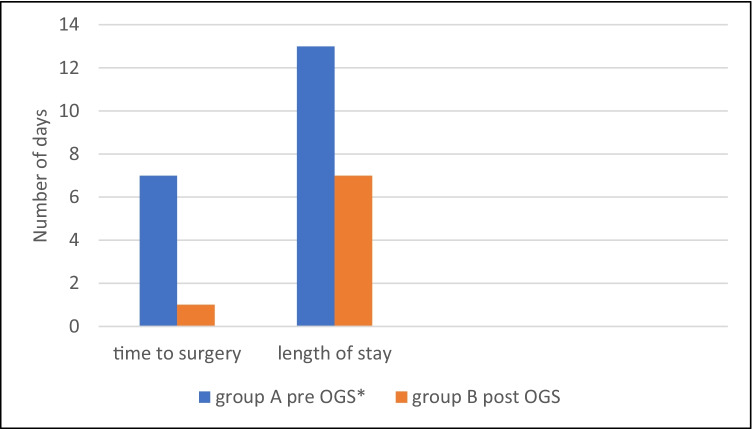
Comparison between the time to surgery and length of stay in Groups A and B. *OGS, orthogeriatric service

Improvement was observed in the median number of days until surgery in post-OGS Group B, which showed a significant overall reduction (*p* < 0.001) (Table 1). This was particularly the case with ankle and hip fractures (*p* = 0.018 and *p* < 0.001, respectively) (Table 4). There was also a significant decrease in the mean length of stay (days) with *p* < 0.001 (Table 1; Fig. 1).

**Table 4:** Comparison between Groups A and B regarding the time to surgery for each fracture type

	Time to surgery				Mann–Whitney test
	Group A		Group B		
	Median (days)	IQR	Median (days)	IQR	
Ankle fracture	8	7–12	1.5	0.5–3	0.018
Hip fracture	7	5–9	1.0	0–3	< 0.001
Distal femur	6	6–6	6.0	6–6	1.000
Distal radius	8	7–12	4.0	0–8	0.169
Femur shaft	6	6–10	5.5	1–10	0.761
Proximal humerus	8	7–8	0.0	0–0	0.099

*IQR*, interquartile range

## Discussion

This study compared fracture management in older adults before and after implementation of an OGS, a co-managed orthogeriatric service, aimed at collaboratively reaching management decisions and overcome obstacles to prompt operative treatment. The OGS takes pride in being the first organised discipline of collaboration between Orthopaedic Surgery, Geriatric Medicine and Anaesthesiology in Egypt.

When we compared the pre-OGS group to the post-OGS group, there was a significant decrease in the time to surgery and overall length of stay of fractured patients in the latter group by using this approach. Other variables, like treatment choice, presence of pre- and postoperative pain and susceptibility to depression, were unaffected.

Many studies have been conducted over the past few years to examine the effectiveness of the OGS model on improving the health services for geriatric patients in various countries. Research has focused mainly on reducing costs, time to surgery, length of stay, readmission rates, decreasing postoperative complications and improving overall health and quality of life outcomes of older patients post-fracture.

Previously published literature on this topic has mixed views on the effectiveness of OGS. However, there is general agreement that a multi-disciplinary approach involving geriatricians does contribute to improving mortality rates and this is mostly dependent on reducing time to surgery, especially within the first 24 h of presentation [12]. Delaying operative intervention in elderly fracture patients has been demonstrated to increase postoperative mortality [13]. In fact, early surgical intervention has been identified as a significant prognostic factor in the overall outcomes for older patients presenting with fractures [14–16]. One explanation given by a review by Sheehan et al. was that shorter intervals before operative intervention meant a decline in infections, thrombo-embolic events and cardio-respiratory complications, which are all associated with being bed-bound [17].

An earlier study by Koval et al. [18] compared the outcomes of two sets of patients: one set before initiating a multi-disciplinary hospital pathway and the other after the pathway was put in place. Significant reductions in length of stay were reported as well as a decline in mortality whether during the hospital stay or 1 year later. Khasraghi et al. [19], Vidan et al. [20] and later Della Rocca et al. [21] and Leung et al. [22] all also found improvements in patient time to surgery and length of stay with geriatric co-management. A recent study at the University of Rochester, which included a cohort of 758 patients, showed an improvement in all clinical outcome parameters, including a 4.3-day length of stay and a comparable 24.1-h average time to surgery [23]. Improving time to surgery is a vital component of orthogeriatric services. It has been suggested that geriatric collaboration increases the likelihood of surgical treatment as a result of better patient optimisation [19], but this was not found in our study where treatment choice was unaffected.

On the other hand, some studies have found no difference in time to surgery or length of stay when managing patients in an organised OGS [24, 25]. Despite this, Khan et al. [24] acknowledged that the role of the geriatrician in the management of elderly orthopaedic patients cannot be dismissed just because it was not reflected in measurable outcomes in their research. On the contrary, there is no denying that older patients with multiple morbidities will benefit from geriatric co-management. They recommended more extensive research to settle this debate.

During the course of this study, we were able to identify some factors that may explain the lack of improvement in some of the pre-determined quality measures, namely, degree of postoperative pain improvement, depression and choice of treatment modality. Delay in initiating pain control, ideally through an organised pain management team either before or after surgery, is one issue. It must also be noted that prior to geriatric co-management, patients were usually given non-steroidal anti-inflammatories to control their pain. Using PHQ2 screening questions alone is another. This is due to time constraints during assessment, but more in-depth evaluation of depression in patients positive for depression is recommended. Time to hospital was not measured in either group and this may have affected treatment choices.

We were also able to recognise areas for improvement; e.g. reluctance in starting weight-bearing exercises postoperatively and the need to achieve rehabilitative targets in hospital were the main problem areas.

### Strengths and limitations

This study represents the findings of the first orthogeriatric service in Egypt. We aimed to pinpoint the strengths of applying these principles to the services offered to older patients and identify any weaknesses. Our work was limited by the relatively small sample size. We were unable to report on short- and long-term patient mortality due to non-compliance to follow-up post-discharge.

## Conclusion

Since the beginning of the OGS, preliminary data have been encouraging, with significant reductions in time to surgery and length of stay. This, along with preoperative medical optimisation and collaborative discharge recommendations, has improved overall patient outcomes even though more research is needed.
